# Comorbidities in Patients with Acute and Chronic Temporomandibular Disorders: An Exploratory Retrospective Study with a Conceptual Interdisciplinary Assessment Framework

**DOI:** 10.3390/healthcare14182939

**Published:** 2026-09-10

**Authors:** Manuela Lalu, Marius Sorin Pop, Dana Carmen Zaha

**Affiliations:** 1 Doctoral School of Biomedical Sciences, University of Oradea, 410087 Oradea, Romania; 2 Pelican Hospital, 410469 Oradea, Romania; sorin.pop@spitalpelican.ro; 3 Department of Preclinical Discipline, Faculty of Medicine and Pharmacy, University of Oradea, 410087 Oradea, Romania; dzaha@uoradea.ro

**Keywords:** temporomandibular disorders, chronic pain, comorbidities, interdisciplinary assessment, kinesiophobia, musculoskeletal pain

## Abstract

Background/Objectives: Temporomandibular disorders (TMDs) affect the masticatory muscles, temporomandibular joints, and related tissues and are frequently associated with pain and functional limitation. Patients with chronic TMD may present multiple comorbidities that complicate clinical management and contribute to symptom persistence. This study aimed to identify comorbid conditions associated with chronic TMD, compare their distribution between chronic and acute TMD presentations, and propose a conceptual interdisciplinary assessment framework. Methods: A retrospective observational study included 82 patients with TMD and at least one symptomatic or documented comorbidity identified during interdisciplinary clinical assessment or within the previous three months. Of these, 58 patients had chronic TMD and 24 had acute TMD. Categorical variables were compared using Fisher’s exact test, with odds ratios and 95% confidence intervals calculated for each comparison. False discovery rate (FDR) correction was applied, and age- and sex-adjusted Firth-penalized logistic regression models were also fitted. Results: In direct group comparisons, chronic TMD was associated with higher frequencies of primary headache, secondary headache, neck pain, lower back pain, endocrine/hormonal disorders, Axis II findings, and kinesiophobia. After age- and sex-adjusted Firth-penalized logistic regression analyses and FDR correction, primary headache, secondary headache, neck pain, lower back pain, endocrine/hormonal disorders, and kinesiophobia remained significantly associated with chronic TMD. No significant differences were observed for sleep bruxism, awake bruxism, or poor sleep quality. Conclusions: Given the retrospective design and limited sample size, the results should be interpreted as exploratory associations rather than causal relationships. Further prospective studies are needed to evaluate and validate the proposed framework and assess its clinical applicability.

## 1. Introduction

Temporomandibular disorders (TMDs) represent a group of conditions affecting the masticatory muscles, temporomandibular joints, and associated structures, and are considered the most common form of non-dental orofacial pain (OFP) [1]. Globally, the reported prevalence of TMDs is approximately 34%, with the highest prevalence observed among individuals aged between 18 and 60 years. Females are consistently more frequently affected than males, although the reported sex differences vary across populations [2,3]. Since the introduction of the Research Diagnostic Criteria for Temporomandibular Disorders (RDC/TMD) and, subsequently, the Diagnostic Criteria for Temporomandibular Disorders (DC/TMD), TMDs have been consistently classified as musculoskeletal conditions based on their clinical and functional characteristics [4].

The International Association for the Study of Pain (IASP) defines pain as “an unpleasant sensory and emotional experience associated with, or resembling that associated with, actual or potential tissue damage” [5]. While acute pain is recognized as a protective mechanism of the central nervous system, typically linked to tissue damage, chronic pain requires a broader conceptual framework. In this context, the clarifying positions of the International Association for the Study of Pain (IASP), as reflected in the current classification developed in collaboration with the WHO and incorporated into the ICD-11, provide the most authoritative terminology and diagnostic guidelines for distinguishing chronic pain as a disease entity in its own right [5,6]. Chronic pain is defined as pain persisting or recurring for longer than three months, and its pathophysiological mechanisms may no longer be directly related to the initial tissue injury that triggered the acute phase [7].

The recent literature has increasingly focused on the central mechanisms potentially involved in the chronicity of TMDs. Svensson et al. reported that patients with chronic OFP may exhibit alterations in nociceptive transmission, pain processing, and inhibitory modulation, highlighting the complex multidimensional nature of chronic pain [8]. An increasing number of studies have reported associations between chronic TMDs and various comorbid conditions [9,10]. Although causal relationships remain incompletely understood, the coexistence of multiple painful or systemic conditions has been frequently observed in patients with chronic TMD and may be relevant in the clinical characterization of these patients [10,11,12,13,14].

Several studies have described different categories of comorbidities associated with TMDs, each presenting distinct clinical particularities that may require careful interdisciplinary evaluation [15]. Among the most frequently reported comorbidities are headaches, cervical pain, lower back pain [16], anxiety, depression, fibromyalgia, and sleep disorders [13]. However, the mechanisms underlying these associations remain incompletely elucidated.

Recent evidence has also demonstrated a substantial overlap between TMDs and various headache disorders, which may complicate both diagnosis and clinical management [17]. The prevalence of headache among patients with TMD has been reported to be approximately 60%, with migraine representing the most frequent, followed by tension-type headaches (about 19%) [18,19,20,21].

In addition, previous studies have reported associations between TMDs and lower back pain [22,23,24], another musculoskeletal condition commonly characterized by persistent pain. A higher prevalence of TMD has been observed among individuals with a history of lower back pain compared to those without such a history [25].

The current literature also suggests significant associations between TMDs and psychological and behavioural factors [26,27,28]. The coexistence of anxiety or depressive symptoms with TMDs may be associated with increased symptom burden and may influence treatment response. Sleep disturbances have likewise been frequently reported in patients with TMD and are considered relevant clinical factors in the persistence of TMD-related symptomatology [29,30,31].

Another group of comorbidities described in association with TMD includes endocrine and hormonal disorders, such as polycystic ovary syndrome and thyroid disorders, conditions that may also be associated with pain modulation and sleep alterations [32,33,34,35,36].

Although numerous studies have described individual comorbidities associated with temporomandibular disorders, relatively little attention has been given to how these findings can be integrated into a structured interdisciplinary clinical assessment. In routine practice, patients with chronic TMD frequently require evaluation by professionals from multiple disciplines, including dentistry, physiotherapy, neurology, psychology, pain medicine, and endocrinology. However, currently available diagnostic recommendations primarily focus on individual disorders rather than providing an integrated conceptual approach for organizing the assessment of multiple coexisting conditions. Therefore, beyond investigating the distribution of selected disorders—namely headache disorders, musculoskeletal pain, endocrine/hormonal disorders, and psychological–behavioural factors, which were selected because they represent the most consistently reported and clinically relevant comorbidity domains associated with TMD in the current literature—the present study also aimed to develop a conceptual interdisciplinary assessment framework intended to facilitate clinical reasoning, interdisciplinary communication, and to generate hypotheses for future prospective research. The proposed framework is not intended to represent a validated clinical algorithm but rather a hypothesis-generating conceptual model informed by the existing literature and organized around the predefined clinical domains investigated in the present retrospective study.

The primary aim of this study was to identify and analyze selected comorbid conditions and compare their distribution between chronic and acute TMD presentations within a clinical cohort of patients with at least one documented comorbidity. A secondary aim was to develop a hypothesis-generating conceptual interdisciplinary assessment framework intended to organize the predefined clinical domains investigated in the present study and to provide a basis for future prospective research.

## 2. Materials and Methods

### 2.1. Study Design and Participants

**Figure 1:**
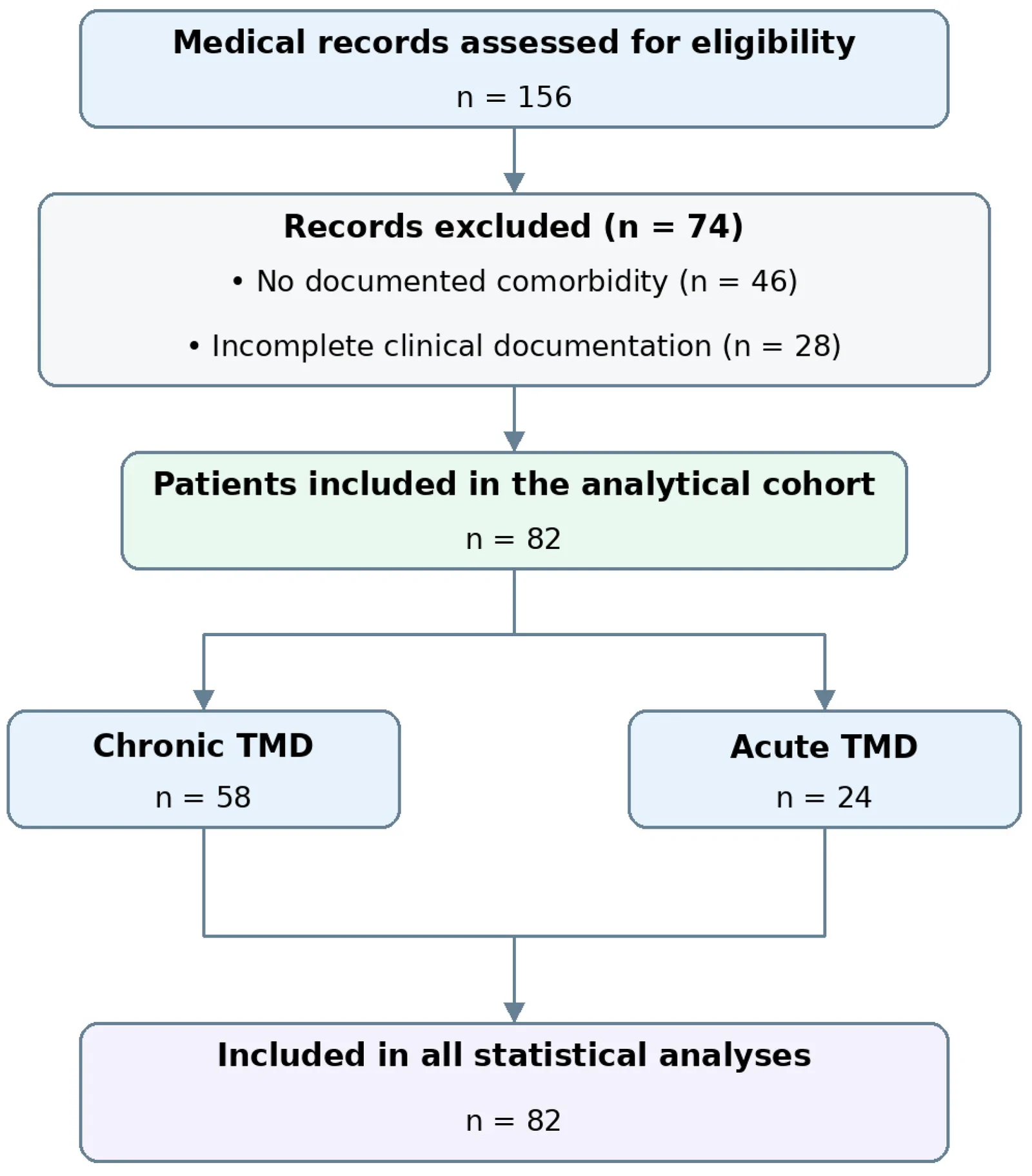
Flow diagram of medical-record screening, eligibility assessment, exclusions, and inclusion in the analytical cohort.

A retrospective observational study based on a medical-record review was conducted between March 2024 and March 2025 at the Physiotherapy Centre of Pelican Hospital, Oradea. During the study period, 156 medical records of patients diagnosed with temporomandibular disorders (TMDs) were screened for eligibility. Seventy-four records were excluded: 46 because no associated comorbidity was documented and 28 because the available clinical documentation was insufficient to confirm eligibility or extract the predefined study variables. Consequently, the final analytical cohort comprised 82 patients with at least one documented comorbidity, including 58 patients with chronic TMD and 24 patients with acute TMD (Figure 1).

Patients were not prospectively recruited. Instead, all available medical records from the predefined study period were reviewed, and only those fulfilling the eligibility criteria were included in the final analysis. Written informed consent for the anonymous use of clinical data for research purposes had been obtained from all patients at the time of hospital admission.

For the purposes of this study, chronicity referred specifically to TMD-related pain duration according to the Diagnostic Criteria for Temporomandibular Disorders (DC/TMD) and the ICD-11 conceptualization of chronic pain. Patients reporting pain persisting or recurring for more than three months were classified as having chronic TMD, whereas those with symptom duration below three months were classified as having acute TMD.

The inclusion criteria were confirmation of TMD according to the DC/TMD, presence of at least one symptomatic or clinically documented comorbidity identified at the time of examination or within the previous three months, availability of complete clinical documentation, and written informed consent for the use of anonymized medical data.

The inclusion of only patients with at least one documented comorbidity was predefined because the objective of the study was to compare the distribution of selected comorbidities between chronic and acute TMD within a clinically relevant comorbidity cohort rather than to estimate the prevalence of comorbidities among all patients with TMD. Consequently, the reported associations should be interpreted within this selected clinical population and should not be generalized to the overall TMD population.

The exclusion criteria were the absence of documented associated comorbidities or insufficient clinical documentation to confirm eligibility or extract the predefined study variables.

### 2.2. Clinical Assessment and Data Extraction

Data were extracted retrospectively by two authors (M.L. and M.S.P.) from the standardized medical records available for each patient. The predefined variables included demographic characteristics, TMD diagnosis, duration of TMD-related pain, documented comorbid conditions, and relevant clinical-functional assessment findings. Comorbidities were identified through retrospective review of the available clinical documentation using predefined extraction criteria rather than through prospective assessment specifically conducted for the purposes of this study.

All patients had undergone clinical evaluation according to the Diagnostic Criteria for Temporomandibular Disorders (DC/TMD) assessment protocol [4]. The clinical examination included standardized assessment of pain history, pain location, familiar pain, mandibular function, and the clinical findings required for Axis I diagnostic classification. The diagnosis of TMD and the corresponding DC/TMD Axis I classification were established by the referring dentist according to the standardized DC/TMD protocol.

The identification of comorbid conditions was based on the source and type of clinical information available in the medical records. Headache disorders, including migraine, tension-type headache, cervicogenic headache, and headache attributed to TMD, were recorded only when a previous neurologist-confirmed diagnosis was documented. Endocrine and hormonal disorders, including thyroid disorders and polycystic ovary syndrome, were considered present only when a diagnosis established by an appropriate medical specialist was available in the patient’s clinical documentation. Gastrointestinal disorders were considered present only when a previously established medical diagnosis was documented in the patient’s clinical record. In the present cohort, this category comprised irritable bowel syndrome, gastritis with gastroesophageal reflux, and celiac disease. These conditions were recorded as pre-existing gastrointestinal comorbidities and were not diagnosed on the basis of the physiotherapy assessment performed in the present study.

For descriptive analyses, “Axis II findings” referred to the presence of clinically relevant anxiety and/or depressive symptoms according to predefined questionnaire thresholds. Anxiety and depressive symptoms were assessed using the validated Romanian-language versions of the Generalized Anxiety Disorder-7 (GAD-7) and Patient Health Questionnaire-9 (PHQ-9), respectively. Scores of ≥10 on either questionnaire were considered indicative of clinically relevant anxiety or depressive symptoms, in accordance with the original validation studies.

Kinesiophobia was assessed using the Tampa Scale for Kinesiophobia for Temporomandibular Disorders (TSK-TMD). A Romanian translation prepared for clinical use was administered consistently to all patients. Although formal psychometric validation of the Romanian version has not yet been completed, the same translated instrument and the predefined threshold of ≥23 were applied uniformly throughout the study.

Awake and sleep bruxism were analyzed as behavioural factors rather than as independent comorbid conditions. They were classified according to current international recommendations for possible bruxism using standardized self-reported assessment and Oral Behaviors Checklist findings as supportive information rather than as definitive diagnostic tests. Sleep quality was assessed using the Pittsburgh Sleep Quality Index (PSQI), and poor sleep quality was classified according to the PSQI scoring criteria applied in the clinical records.

Musculoskeletal comorbidities, including neck pain and lower back pain, were based on previous specialist-confirmed clinical diagnoses recorded in the medical files and were complemented by standardized functional assessment performed by two authors (M.L. and M.S.P.), both physiotherapists. All patients underwent the same interdisciplinary clinical assessment irrespective of TMD duration. Because this was a retrospective medical-record review, assessor blinding to symptom duration was not applicable. Formal inter-rater reliability was not evaluated; however, both physiotherapists followed the same standardized assessment protocol and predefined examination procedures throughout the study.

For patients presenting with musculoskeletal symptoms, the cervical spine assessment included muscle palpation for familiar pain reproduction, the Spurling test, cervical traction and stretch tests, the Jackson compression test, the Adson test, and the Flexion Rotation Test. Lumbar assessment included the Oswestry Disability Index, active range-of-motion evaluation, and Movement Impairment Syndrome assessment according to the principles described by Shirley A. Sahrmann.

The results of individual clinical-functional tests were used to support standardized physiotherapy assessment and clinical reasoning but were not analyzed as independent study variables. For statistical analysis, only the final predefined binary variables representing the presence or absence of musculoskeletal comorbidities, including neck pain and lower back pain, were entered into the dataset.

Because the investigated comorbidities were identified from different components of routine clinical care—including previous specialist diagnoses, medical records, standardized questionnaires, and interdisciplinary clinical assessment—some variability in diagnostic ascertainment may have been present. Nevertheless, predefined operational criteria and standardized extraction procedures were applied consistently to all eligible records.

### 2.3. Development of the Conceptual Interdisciplinary Assessment Framework

To facilitate the interdisciplinary interpretation of the collected clinical information, the investigated comorbidities were organized into predefined clinical domains according to their principal clinical characteristics and the medical specialty most commonly involved in their assessment. This conceptual organization was developed by the authors for descriptive and analytical purposes to support the presentation and interpretation of the study findings.

The proposed framework comprised four predefined domains:

- Musculoskeletal-related comorbidities, including neck pain, lower back pain, and other musculoskeletal pain conditions identified during clinical assessment or documented in previous medical records;
- Psychological and behavioural factors, including anxiety, depressive symptoms, kinesiophobia, awake bruxism, sleep bruxism, poor sleep quality, and other relevant behavioural manifestations assessed using standardized clinical instruments;
- Headache-related disorders, including primary headaches (migraine and tension-type headache) and secondary headaches (cervicogenic headache and headache attributed to TMD), recorded only when documented by a neurologist according to established diagnostic criteria;
- Systemic comorbidities, consisting primarily of endocrine and hormonal disorders, including thyroid disorders and polycystic ovary syndrome, identified from previous specialist diagnoses documented in the medical records.

Based on this organization, the authors developed a conceptual clinical-reasoning framework in which patients with confirmed TMD are first considered according to TMD-related pain duration, distinguishing acute from chronic presentations. For patients with chronic TMD, the framework proposes the structured consideration of four clinical domains investigated in the present study: headache-related disorders, musculoskeletal comorbidities, psychological–behavioural factors, and systemic endocrine/hormonal disorders. These domains are intended to organize clinical reasoning and to identify areas that may merit interdisciplinary assessment when clinically relevant. The transition from the empirical findings to this structured clinical-reasoning pathway represents an author-derived conceptual proposal and was not prospectively evaluated in the present study. Accordingly, the framework should not be interpreted as a validated diagnostic, referral, or treatment algorithm, and its potential clinical utility requires prospective evaluation and validation.

### 2.4. Statistical Analysis

Statistical analyses were performed using IBM SPSS Statistics for Windows, version 24 (IBM Corp., Armonk, NY, USA). Because this was a retrospective observational study based on all eligible medical records available during a predefined study period, no a priori sample size or power calculation was performed. The final sample size was therefore determined by the number of records meeting the predefined eligibility criteria.

Continuous variables were summarized as mean ± standard deviation (SD), whereas categorical variables were presented as absolute frequencies and percentages. Differences in age between the chronic and acute TMD groups were evaluated using Welch’s *t*-test because of unequal group sizes. Categorical variables were compared using Fisher’s exact test owing to the relatively small sample size and the presence of low expected cell counts.

For each categorical comparison, odds ratios (ORs) with 95% confidence intervals (95% CIs) were calculated to estimate the magnitude of the observed associations. For 2 × 2 contingency tables containing zero-cell counts, ORs and corresponding 95% CIs were estimated using the Haldane–Anscombe continuity correction by adding 0.5 to each cell. Fisher’s exact test was used to calculate the corresponding *p*-values and was not affected by this continuity correction.

For the predefined direct group comparisons, the Benjamini–Hochberg false discovery rate (FDR) correction was applied across the 11 Fisher’s exact-test *p*-values as a single family of comparisons. Separately, for the age- and sex-adjusted Firth-penalized logistic regression analyses, the Benjamini–Hochberg procedure was applied across the 11 penalized likelihood-ratio *p*-values. Thus, multiplicity adjustment was performed separately for the unadjusted direct comparisons and the adjusted regression analyses.

Age- and sex-adjusted Firth-penalized logistic regression models constituted the principal inferential analyses. Separate models were fitted for each predefined comorbidity, with chronic TMD status as the dependent variable. Firth-penalized logistic regression was selected because of the relatively small sample size and sparse data observed for several variables, thereby reducing the risk of small-sample bias and separation. Separate models were fitted because the available sample size, particularly in the acute TMD group, did not permit construction of a stable multivariable model including all predefined comorbidity variables simultaneously. The unadjusted Fisher’s exact tests were retained to describe the direct group comparisons. Given the limited sample size and sparse event counts, the adjusted analyses were nevertheless considered exploratory and hypothesis-generating rather than confirmatory.

Only patients with complete clinical documentation fulfilling the predefined eligibility criteria were included in the final analytical cohort; therefore, no missing data were present and no imputation procedures were required.

For the predefined direct group comparisons, statistical significance was defined as *p* < 0.05. Following correction for multiple comparisons, FDR-adjusted *p*-values < 0.05 were considered statistically significant. Unless otherwise specified, the interpretation of the predefined comorbidity analyses was based primarily on the FDR-adjusted *p*-values, whereas the corresponding unadjusted *p*-values are also reported to facilitate comparison with the previous literature. Given the retrospective observational design of the study, all reported associations were interpreted as statistical associations rather than evidence of causal relationships.

## 3. Results

The final analytical cohort comprised 82 patients, including 58 patients with chronic TMD and 24 patients with acute TMD. The demographic characteristics of the study groups are summarized in Table 1. Female patients represented 89.7% of the chronic TMD group and 62.5% of the acute TMD group. The difference in sex distribution between the two groups was statistically significant (OR = 5.20, 95% CI: 1.59–16.96; *p* = 0.009). No statistically significant difference in age was observed between the two groups.

Primary headache disorders were identified in 48.3% (*n* = 28) of patients in the chronic TMD group, compared with 16.7% (*n* = 4) of patients in the acute TMD group. This difference was statistically significant after FDR correction (OR = 4.67, 95% CI: 1.42–15.35; Fisher *p* = 0.012; FDR *p* = 0.019).

Secondary headaches were present in 37.9% (*n* = 22) of chronic TMD patients and in 4.2% (*n* = 1) of acute TMD patients. This association also remained statistically significant after FDR correction (OR = 14.06, 95% CI: 1.77–111.52; Fisher *p* = 0.002; FDR *p* = 0.006). Overall, headache-related comorbidities were more frequently observed in patients with chronic TMD than in those with acute TMD.

The headache-related comorbidities in the chronic and acute TMD groups are summarized in Table 2.

Musculoskeletal-related comorbidities were more frequently observed in the chronic TMD group. Neck pain was identified in 43.1% (*n* = 25) of patients with chronic TMD, compared with 8.3% (*n* = 2) of patients with acute TMD. This difference was statistically significant after FDR correction (OR = 8.33, 95% CI: 1.79–38.79; Fisher *p* = 0.002; FDR *p* = 0.006).

Lower back pain was also more frequent in the chronic TMD group, being present in 43.1% (*n* = 25) of chronic TMD patients and in 8.3% (*n* = 2) of acute TMD patients. This association remained statistically significant after FDR correction (OR = 8.33, 95% CI: 1.79–38.79; Fisher *p* = 0.002; FDR *p* = 0.006).

After adjustment for age and sex using adjusted Firth-penalized logistic regression, neck pain and lower back pain remained significantly associated with chronic TMD.

The musculoskeletal-related comorbidities in the chronic and acute TMD groups are summarized in Table 3.

Regarding systemic comorbidities, documented gastrointestinal disorders were identified only in the chronic TMD group, being present in 15.5% (*n* = 9) of patients with chronic TMD and in none of the patients with acute TMD. Among these nine patients, four had irritable bowel syndrome, two had gastritis with gastroesophageal reflux, and three had celiac disease. Although this difference showed an exploratory association in the direct group comparisons, it did not remain statistically significant after FDR correction (OR = 9.40, 95% CI: 0.53–168.33; Fisher *p* = 0.053; FDR *p* = 0.072).

Endocrine/hormonal disorders were more frequently reported among patients with chronic TMD than among those with acute TMD. Among the 26 patients with endocrine/hormonal disorders in the chronic TMD group, 15 had polycystic ovary syndrome, 10 had thyroid disorders, and one patient presented with both conditions. In the acute TMD group, two patients had thyroid disorders and one patient presented with both thyroid disease and polycystic ovary syndrome. These disorders were identified in 44.8% (*n* = 26) of patients in the chronic TMD group and in 12.5% (*n* = 3) of patients in the acute TMD group. This association remained statistically significant after FDR correction (OR = 5.69, 95% CI: 1.53–21.20; Fisher *p* = 0.005; FDR *p* = 0.011).

The systemic comorbidities in the chronic and acute TMD groups are summarized in Table 4.

Psychological and behavioural factors were also assessed. Axis II findings were identified in 69.0% (*n* = 40) of patients with chronic TMD and in 33.3% (*n* = 8) of patients with acute TMD. This difference was statistically significant after FDR correction (OR = 4.44, 95% CI: 1.61–12.26; Fisher *p* = 0.006; FDR *p* = 0.011).

Kinesiophobia remained significantly associated with chronic TMD after adjustment for age and sex in the Firth-penalized logistic regression analysis and FDR correction.

No statistically significant differences were observed between the chronic and acute TMD groups regarding sleep bruxism, awake bruxism, or poor sleep quality. Sleep bruxism was identified in 29.3% (*n* = 17) of chronic TMD patients and 37.5% (*n* = 9) of acute TMD patients. Awake bruxism was present in 44.8% (*n* = 26) of chronic TMD patients and 50.0% (*n* = 12) of acute TMD patients. Poor sleep quality was reported in 41.4% (*n* = 24) of chronic TMD patients and 37.5% (*n* = 9) of acute TMD patients.

After adjustment for age and sex using Firth-penalized logistic regression, kinesiophobia remained significantly associated with chronic TMD, suggesting that this was one of the most consistent psychological–behavioural findings of the present study.

The psychological and behavioural factors in the chronic and acute TMD groups are summarized in Table 5.

After adjustment for age and sex using Firth-penalized logistic regression models, primary headache, secondary headache, neck pain, lower back pain, endocrine/hormonal disorders, and kinesiophobia remained significantly associated with chronic TMD. Axis II findings and gastrointestinal disorders did not reach the FDR-adjusted significance threshold.

**Table 6:** Age- and sex-adjusted associations between predefined comorbidities and chronic TMD estimated using Firth-penalized logistic regression.

Variable	Chronic	Acute	Adjusted OR	95% CI	*p*-Value	FDR-Adjusted *p*-Value
Primary headache	28/58 (48.3%)	4/24 (16.7%)	3.27	1.04–12.09	0.021	0.039
Secondary headache	22/58 (37.9%)	1/24 (4.2%)	7.27	1.65–68.64	0.004	0.010
Neck pain	25/58 (43.1%)	2/24 (8.3%)	6.68	1.82–36.74	0.001	0.005
Lower back pain	25/58 (43.1%)	2/24 (8.3%)	8.63	2.20–50.86	<0.001	0.003
Gastrointestinal disorders	9/58 (15.5%)	0/24 (0.0%)	8.77	0.94–1185.23	0.049	0.067
Endocrine/hormonal disorders	26/58 (44.8%)	3/24 (12.5%)	3.74	1.10–16.09	0.019	0.039
Axis II findings	40/58 (69.0%)	8/24 (33.3%)	2.82	0.95–8.56	0.032	0.051
Sleep bruxism	17/58 (29.3%)	9/24 (37.5%)	1.14	0.39–3.71	0.283	0.283
Awake bruxism	26/58 (44.8%)	12/24 (50.0%)	1.43	0.50–4.45	0.201	0.221
Kinesiophobia	34/58 (58.6%)	2/24 (8.3%)	10.11	2.84–53.84	<0.001	<0.001
Poor sleep quality	24/58 (41.4%)	9/24 (37.5%)	1.59	0.57–4.81	0.154	0.188

Note. OR, odds ratio; CI, confidence interval; FDR, false discovery rate. Separate Firth-penalized logistic regression models were fitted for each predefined comorbidity, with chronic TMD status as the dependent variable and adjustment for age and sex. Profile likelihood 95% confidence intervals and penalized likelihood-ratio *p*-values are reported. FDR-adjusted *p*-values were calculated using the Benjamini–Hochberg procedure across the 11 predefined comorbidity models.

## 4. Discussion

The present study explored the association between chronic temporomandibular disorders (TMDs) and several categories of comorbid conditions identified in a retrospective clinical cohort. Compared with patients presenting with acute TMD, individuals with chronic TMD exhibited a higher frequency of headache-related disorders, musculoskeletal pain conditions, endocrine/hormonal disorders, and psychological–behavioural factors. After adjustment for age and sex using Firth-penalized logistic regression with false discovery rate correction, primary headache, secondary headache, neck pain, lower back pain, endocrine/hormonal disorders, and kinesiophobia remained significantly associated with chronic TMD, whereas Axis II findings and gastrointestinal disorders did not retain statistical significance. These findings support the concept that chronic TMD frequently occurs within a broader multidimensional clinical context extending beyond the temporomandibular region.

The association observed between chronic TMD and headache disorders is consistent with previous reports describing a substantial overlap between temporomandibular disorders and both primary and secondary headache syndromes [17,19,21,37]. Patients with chronic TMD more frequently presented with migraine, tension-type headache, cervicogenic headache, and headache attributed to TMD than patients with acute TMD. Importantly, these associations remained statistically significant after adjustment for age and sex, suggesting that headache disorders represent one of the findings that remained statistically significant in the exploratory adjusted analyses. This finding is also consistent with recent meta-analytic evidence showing an increased occurrence of TMD among patients with migraine and tension-type headache [37]. Although the present study cannot establish temporal or causal relationships, the findings reinforce previous evidence supporting a close clinical interaction between chronic orofacial pain and headache disorders and suggest that headache-related symptoms represent a relevant domain for further interdisciplinary investigation in patients with TMD.

Musculoskeletal comorbidities demonstrated a similarly consistent pattern. Neck pain and lower back pain were both more frequent among patients with chronic TMD and remained significantly associated with chronic TMD after adjustment for age and sex. These observations agree with previous studies suggesting that chronic musculoskeletal pain conditions frequently coexist and may share mechanisms involving altered pain modulation, central sensitization, or common biomechanical and behavioural factors [16,23,24,25,38,39]. In particular, recent systematic-review and meta-analytic evidence has supported an association between low back pain and painful TMD, including an increased likelihood of chronic TMD among individuals with chronic low back pain [38]. These findings suggest that musculoskeletal features may represent a relevant domain for further investigation in patients with persistent TMD symptoms.

Psychological and behavioural factors also demonstrated clinically relevant associations. Anxiety and depressive symptoms were more frequent in patients with chronic TMD in the unadjusted analyses, although this association no longer remained statistically significant after age- and sex-adjusted analyses with FDR correction. Nevertheless, the higher unadjusted frequency observed in the chronic TMD group is compatible with longitudinal evidence identifying anxiety and depressive symptoms among factors associated with TMD development [27,28,40,41,42]. In contrast, kinesiophobia remained statistically significant in the exploratory age- and sex-adjusted analyses. This finding supports the growing recognition that fear of movement represents an important component of chronic musculoskeletal pain conditions and may contribute to disability, avoidance behaviours, and persistence of symptoms. Recent systematic-review evidence specifically addressing TMD also supports the clinical relevance of kinesiophobia and pain catastrophizing in TMD-related pain and disability [43]. Conversely, no statistically significant differences were identified for awake bruxism, sleep bruxism, or poor sleep quality. However, given the limited sample size and statistical power, these findings should not be interpreted as evidence of equivalence between the acute and chronic TMD groups. These findings should also be interpreted in the context of the previous literature reporting associations between sleep-related factors and TMD [30,31,32,42,44], suggesting that the absence of statistically significant differences in the present cohort does not necessarily indicate the absence of a clinically relevant relationship. Larger studies are required to determine whether smaller differences in these behavioural and sleep-related factors exist.

Endocrine and hormonal disorders also remained significantly associated with chronic TMD after adjustment for age and sex. Although the biological mechanisms underlying these associations remain incompletely understood, the previous literature has suggested potential interactions involving hormonal regulation, pain modulation, inflammatory pathways, and sleep disturbances [33,34,35,36,45,46]. The present findings are therefore broadly compatible with previous reports linking TMD with endocrine and hormonal conditions, although the current data do not permit conclusions regarding the direction or biological mechanisms of these associations [33,34,35,36,45,46]. These findings should nevertheless be interpreted cautiously because of the relatively small number of patients within individual diagnostic categories.

Collectively, these findings emphasize that patients with chronic TMD may present with multiple coexisting conditions involving different clinical domains, consistent with the multidimensional pattern of factors reported in recent systematic-review evidence [10,15,42]. For this reason, isolated assessment focused exclusively on the temporomandibular joint may fail to capture clinically relevant aspects of the patient’s overall condition. These findings highlight the potential relevance of considering multiple clinical domains when characterizing patients with persistent TMD symptoms, while the clinical utility of a structured interdisciplinary assessment approach requires prospective evaluation.

**Figure 2:**
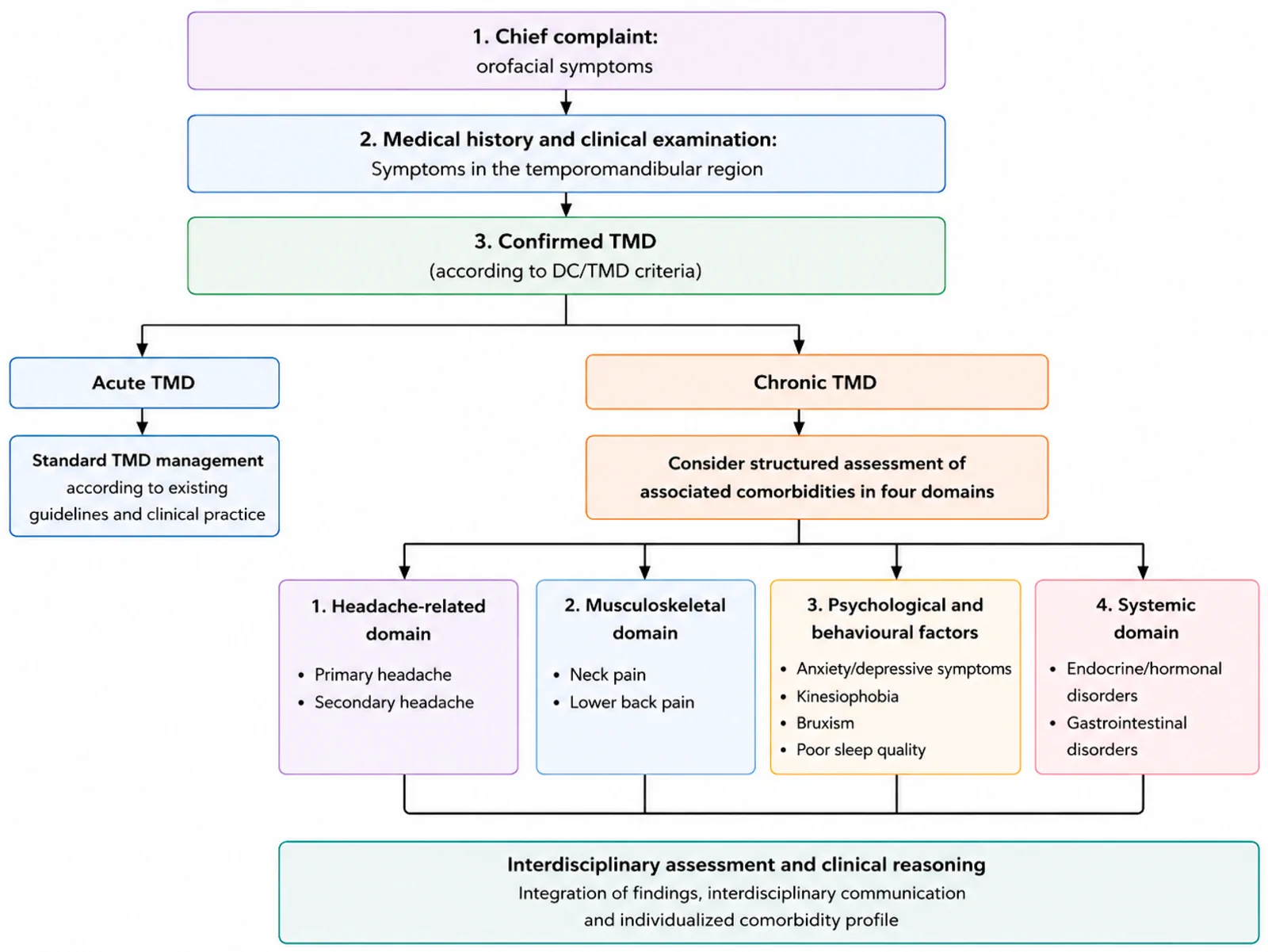
Proposed conceptual clinical-reasoning framework for patients with chronic temporomandibular disorders Note. Author-derived conceptual clinical-reasoning framework for patients with chronic temporomandibular disorders (TMDs) and associated comorbidities. The framework organizes the clinical domains investigated in the present study to support structured interdisciplinary consideration. It has not undergone formal consensus development or prospective validation and should not be interpreted as a diagnostic, referral, treatment, or clinical decision-making algorithm.

To translate these observations into a structured conceptual approach to clinical reasoning, the present study proposes an interdisciplinary framework that begins with confirmed TMD and distinguishes acute from chronic presentations according to TMD-related pain duration. In patients with chronic TMD, the framework proposes structured consideration of four clinical domains investigated in the present study: headache-related disorders, musculoskeletal comorbidities, psychological–behavioural factors, and systemic endocrine/hormonal disorders. The purpose of this structure is not to prescribe diagnostic, referral, or treatment decisions, but to highlight clinical domains that may merit consideration during interdisciplinary assessment and clinical reasoning, according to the individual patient presentation. The transition from the observed comorbidity patterns to this structured pathway represents an author-derived conceptual proposal and was not evaluated as an intervention in the present retrospective study. Accordingly, the framework should be regarded as hypothesis-generating and requires prospective evaluation to determine its reproducibility, clinical utility, and potential contribution to interdisciplinary assessment. The proposed conceptual clinical-reasoning framework is illustrated in Figure 2.

### 4.1. Limitations

Several limitations should be considered when interpreting the present findings. First, the retrospective observational design precludes conclusions regarding causality or temporal relationships between TMD chronicity and the investigated comorbidities. Because TMD-related pain duration and comorbid conditions were assessed retrospectively over overlapping time periods, their temporal sequence cannot be established, and reverse temporality cannot be excluded. Patients with chronic TMD may also have had greater healthcare utilization and more opportunities for clinical investigation and documentation of associated conditions than patients with recent-onset TMD, potentially introducing surveillance (ascertainment) bias. Second, the relatively limited sample size, particularly in the acute TMD group, reduced statistical power and precluded construction of a stable multivariable model including all predefined variables simultaneously. Although separate age- and sex-adjusted Firth-penalized logistic regression models were used to reduce small-sample bias and potential confounding, these analyses remain exploratory, and residual confounding by TMD subtype, symptom severity, pain intensity, healthcare utilization, psychological distress, previous treatments, medication use, and other unmeasured clinical variables cannot be excluded.

Third, comorbidities were identified from different components of routine clinical care, including previous specialist-confirmed diagnoses, standardized questionnaires, and interdisciplinary clinical assessments. Although predefined operational criteria and standardized data-extraction procedures were consistently applied, variability in diagnostic ascertainment cannot be excluded. Moreover, assessor blinding was not applicable and formal inter-rater reliability was not evaluated; therefore, investigator-led components of the clinical assessment may have been subject to observer or assessor bias. Fourth, only patients with at least one documented comorbidity were eligible for inclusion, potentially introducing selection bias and influencing the observed distribution and magnitude of associations. Accordingly, the study represents a selected clinical cohort; the findings should not be interpreted as prevalence estimates for the overall TMD population; and the single-centre setting further limits external generalizability.

Fifth, although TMD diagnosis was established according to the DC/TMD protocol, patients were not stratified according to specific DC/TMD Axis I diagnostic subtypes, such as myalgia, arthralgia, disc displacement, or degenerative joint disease. Therefore, the observed associations between TMD-related pain chronicity and comorbidities cannot be attributed to specific TMD subtypes. Sixth, detailed quantitative orofacial functional measures, including maximum mouth opening, mandibular range of motion, deviation during opening, and standardized masticatory muscle and temporomandibular joint palpation findings, were not systematically extracted and analyzed as study variables. Standardized TMJ imaging was also not systematically available; consequently, structural characteristics such as disc position and morphology, joint-space alterations, and degenerative osseous changes could not be evaluated or compared between groups. Finally, the proposed interdisciplinary framework was derived from the present clinical observations and available literature and has not undergone prospective validation or evaluation of its impact on clinical decision-making.

Despite these limitations, the study has several strengths, including standardized DC/TMD-based clinical evaluation, predefined operational criteria for retrospective data extraction, correction for multiple comparisons, and the use of Firth-penalized logistic regression to reduce small-sample bias. The integration of findings across multiple clinical domains into a single conceptual framework also provides a structured basis for future prospective investigation.

### 4.2. Recommendations for Future Research

Future prospective multicentre studies involving larger and more balanced patient populations are needed to confirm the observed associations and investigate their temporal and potential causal relationships. Such studies should incorporate subtype-specific DC/TMD stratification, standardized quantitative orofacial functional assessments, and, where clinically appropriate, standardized TMJ imaging to provide more comprehensive clinical phenotyping. Independent prospective validation of the proposed conceptual framework in well-characterized cohorts will also be necessary to determine its reproducibility and clinical utility before routine implementation can be considered.

## 5. Conclusions

Within this selected clinical cohort, chronic TMD was characterized by a broader pattern of documented comorbidities across headache-related, musculoskeletal, psychological–behavioural, and systemic domains compared with acute TMD. These findings support the relevance of considering TMD-related pain chronicity within a multidimensional clinical context rather than in isolation. However, given the retrospective design, limited sample size, sparse event counts, and potential residual confounding and ascertainment bias, the observed associations should be regarded as exploratory and hypothesis-generating rather than causal or clinically predictive. The proposed interdisciplinary framework provides a conceptual structure for organizing the clinical domains investigated in this study but requires prospective validation before its clinical utility can be established.

## Figures and Tables

**Table 1:** Demographic characteristics of the study groups.

Variable	Type	Chronic (G1)	Acute (G2)	OR (95% CI)	*p*-Value
Sex	Male	6/58 (10.3%)	9/24 (37.5%)	Reference 5.20 (1.59–16.96)	0.009
	Female	52/58 (89.7%)	15/24 (62.5%)		
Age	Mean ± SD	32.21 ± 10.05	35.38 ± 7.65		0.127

Note: SD, standard deviation; OR, odds ratio; CI, confidence interval. Age was compared with Welch’s *t*-test. Sex distribution was compared using Fisher’s exact test. ORs and 95% confidence intervals are reported for categorical variables only.

**Table 2:** Headache-related comorbidities in chronic and acute TMD groups.

Variable	Chronic	Acute	OR (95% CI)	Fisher *p*	FDR *p*
Primary headache	28/58(48.3%)	4/24 (16.7%)	4.67 (1.42–15.35)	0.012	0.019
Secondary headache	22/58 (37.9%)	1/24 (4.2%)	14.06 (1.77–111.52)	0.002	0.006

Note. OR, odds ratio; CI, confidence interval; FDR, false discovery rate (Benjamini–Hochberg correction). Primary and secondary headache were analyzed as separate predefined variables; however, complete mutual exclusivity between these categories could not be established from the retrospective records, and the two headache outcomes should therefore not be interpreted as statistically independent clinical phenomena.

**Table 3:** Musculoskeletal-related comorbidities in chronic and acute TMD groups.

Variable	Chronic	Acute	OR (95% CI)	Fisher *p*	FDR *p*
Neck pain	25/58(43.1%)	2/24 (8.3%)	8.33 (1.79–38.79)	0.002	0.006
Lower back pain	25/58 (43.1%)	2/24(8.3%)	8.33 (1.79–38.79)	0.002	0.006

Note. OR, odds ratio; CI, confidence interval; FDR, false discovery rate (Benjamini–Hochberg correction). In the present cohort, neck pain and lower back pain occurred in the same patients in both the chronic and acute TMD groups; therefore, these two musculoskeletal variables should not be interpreted as independent clinical findings.

**Table 4:** Systemic comorbidities in chronic and acute TMD groups.

Variable	Chronic	Acute	OR (95% CI)	Fisher *p*	FDR *p*
Gastrointestinal disorders	9/58 (15.5%)	0/24 (0.0%)	9.40 (0.53–168.33)	0.053	0.072
Endocrine/hormonal disorders	26/58 (44.8%)	3/24 (12.5%)	5.69 (1.53–21.20)	0.005	0.011

Note. OR, odds ratio; CI, confidence interval; FDR, false discovery rate (Benjamini–Hochberg correction). Gastrointestinal disorders comprised irritable bowel syndrome (*n* = 4), gastritis with gastroesophageal reflux (*n* = 2), and celiac disease (*n* = 3).

**Table 5:** Psychological and behavioural factors in chronic and acute TMD groups.

Variable	Chronic	Acute	OR (95% CI)	Fisher *p*	FDR *p*
AXIS II	40/58 (69.0%)	8/24 (33.3%)	4.44 (1.61–12.26)	0.006	0.011
Sleep bruxism	17/58(29.3%)	9/24 (37.5%)	0.49 (0.18–1.31)	0.203	0.223
Awake bruxism	26/58 (44.8%)	12/24 (50.0%)	0.58 (0.22–1.51)	0.335	0.335
Kinesiophobia	34/58 (58.6%)	2/24 (8.3%)	15.58 (3.34–72.62)	<0.001	<0.001
Poor sleep quality	24/58 (41.4%)	9/24 (37.5%)	0.78 (0.30–2.03)	0.630	0.630

Note. Age- and sex-adjusted associations are presented in Table 6 using Firth-penalized logistic regression models. OR, odds ratio; CI, confidence interval; FDR, false discovery rate (Benjamini–Hochberg correction).

## Data Availability

The data supporting the findings of this study are available from Pelican Hospital upon reasonable request and subject to institutional and ethical restrictions.

## Abbreviations

The following abbreviations are used in this manuscript:

AROM	Active Range of Motion
CI	Confidence Interval
DC/TMDs	Diagnostic Criteria for Temporomandibular Disorders
FDR	False Discovery Rate
OR	Odds Ratio
FRT	Flexion Rotation Test
GAD-7	Generalized Anxiety Disorder-7
ICD-11	International Classification of Diseases, 11th Revision
IASP	International Association for the Study of Pain
MIS	Movement Impairment Syndrome
OBC	Oral Behaviors Checklist
ODI	Oswestry Disability Index
OFP	Orofacial Pain
PHQ-9	Patient Health Questionnaire-9
PSQI	Pittsburgh Sleep Quality Index
RDC/TMDs	Research Diagnostic Criteria for Temporomandibular Disorders
TMDs	Temporomandibular Disorders
TMJ	Temporomandibular Joint
TSK-TMDs	Tampa Scale for Kinesiophobia for Temporomandibular Disorders
